# Lack of germline transmission in male mice following a single intravenous administration of AAV5-hFVIII-SQ gene therapy

**DOI:** 10.1038/s41434-022-00318-5

**Published:** 2022-02-07

**Authors:** Carlos Fonck, Cheng Su, Jeremy Arens, Elli Koziol, Jaydeep Srimani, Joshua Henshaw, Andrea Van Tuyl, Sundeep Chandra, Christian Vettermann, Charles A. O’Neill

**Affiliations:** 1 Pharmacological Sciences, BioMarin Pharmaceutical Inc., Novato, CA USA; 2 Data Sciences and Analytics, BioMarin Pharmaceutical Inc., Novato, CA USA; 3 Clinical Pharmacology, BioMarin Pharmaceutical Inc., Novato, CA USA; 4 BioAnalytical Sciences, BioMarin Pharmaceutical Inc., Novato, CA USA; 5 Pathology, BioMarin Pharmaceutical Inc., Novato, CA USA

**Keywords:** Gene therapy, Gene therapy

## Abstract

Valoctocogene roxaparvovec (AAV5-hFVIII-SQ) is an adeno-associated virus serotype five gene therapy under investigation for the treatment of hemophilia A. Herein, we assessed the potential for germline transmission of AAV5-hFVIII-SQ in mice. Male B6.129S6-*Rag2*^*tm1Fwa*^ N12 mice received a single intravenous dose of vehicle or 6 × 10^13^ vg/kg AAV5-hFVIII-SQ. Vehicle and AAV5-hFVIII-SQ-treated mice were mated with naïve females 4 days after dosing, when the concentration of vector genomes was expected to be at its peak in semen, and 37 days after dosing, when a full spermatogenesis cycle was estimated to be complete. Quantitative PCR was used to evaluate the presence of transgene DNA in liver and testes from F0 males dosed with AAV5-hFVIII-SQ and liver tissue of F1 offspring. Transgene DNA was detected in liver and testes of all F0 males dosed with AAV5-hFVIII-SQ, confirming successful transduction. Importantly, no transgene DNA was detected in any tested F1 offspring derived from F0 males dosed with AAV5-hFVIII-SQ. Using a novel 2-stage statistical model that takes into account the number of males dosed with AAV5-hFVIII-SQ and the number of offspring sired by these males, we estimate that the risk of germline transmission is <5% with a 99.2% confidence level.

## Introduction

Hemophilia A is an X-linked bleeding disorder caused by mutations in the Factor VIII (FVIII) gene that affects approximately 1 in 5000 males worldwide [1]. People with severe hemophilia A (FVIII ≤ 1 IU/dL) are at risk of spontaneous bleeding, joint damage that can result in disabling arthropathy, and in some cases, fatal brain hemorrhages [2, 3]. Valoctocogene roxaparvovec (AAV5-hFVIII-SQ) is an adeno-associated virus serotype 5 (AAV5) vector that contains a coding sequence for the human Factor VIII-SQ (hFVIII-SQ) protein. We previously showed that AAV5-hFVIII-SQ gene therapy results in safe and efficacious expression of hFVIII-SQ protein in mice lacking FVIII and clinical trial participants with severe hemophilia A [4–7].

Systemically-delivered AAV-based gene therapies often result in a wide distribution of vector genomes to multiple tissues, including gonads [8–12]. In clinical trials, residual AAV5-hFVIII-SQ vector DNA was detected in seminal fluid for up to 12 months following a single administration, albeit no vector DNA was detected in sperm cells [6]. Therefore, an important concern regarding AAV-mediated gene transfer for the treatment of somatic diseases is the potential for transgene DNA to be incorporated into the human germline.

Various approaches have been pursued to investigate the potential for AAV-based gene therapies to cause germline transmission, including the detection and quantitation of transgene DNA in the gonads and/or in the offspring of animals that received AAV-based gene therapy [8–17]. In this study, the potential for germline transmission of AAV5-hFVIII-SQ was evaluated in male B6.129S6-*Rag2*^*tm1Fwa*^ N12 mice, which lack the *RAG2* gene necessary for mature T and B lymphocyte production and are thus unable to mount a humoral immune response against the hFVIII-SQ transgene protein [18]. Male mice dosed with AAV5-hFVIII-SQ were bred with naïve females, and offspring were evaluated for the presence of transgene DNA.

This study features a 2-stage statistical model that assumes two distinct events need to occur for successful germline transmission: first, F0 males that received AAV5-hFVIII-SQ acquired and transmitted the transgene DNA to their offspring (F1 generation), and second, F1 mice inherited and retained the AAV5-hFVIII-SQ transgene DNA. This statistical model was first used to perform a power analysis calculation, based on hypothetical probabilities assigned to the two events above, that determined the minimal number of F0 and F1 mice necessary to detect at least one successful germline transmission event. The model was then used to establish a confidence level based on the results to qualify the claim that AAV5-hFVIII-SQ is not transmitted to the next generation via the germline.

## Materials and methods

### Study design

AAV5-hFVIII-SQ is a recombinant, replication-incompetent AAV5 vector that contains a single-stranded, codon-optimized hFVIII-SQ coding sequence controlled by a liver-selective promoter [4, 6]. The concentration of AAV5-hFVIII-SQ dosing solution was determined via a titration method based on quantitative PCR (qPCR). A dose of 6 × 10^13^ vg/kg AAV5-hFVIII-SQ was selected for this study because it was the highest dose utilized in clinical trials [5–7]. The vehicle control consisted of 10 mM sodium phosphate, 140 mM sodium chloride, 2% mannitol, and 0.2% pluronic F-68.

**Fig. 1:**
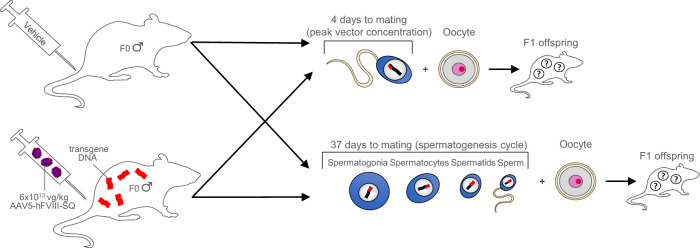
Study design schematic. F0 male mice received an injection of either vehicle or 6 × 10^13^ vg/kg AAV5-hFVIII-SQ and were mated afterward at two different timepoints to naïve females. Some F0 males were mated 4 days afterward, when transgene DNA in semen was estimated to be at peak concentration, and some were mated 37 days after injection, when a full spermatogenesis cycle was expected to complete. F0 males were assessed for transgene DNA in the testes and liver and for hFVIII-SQ protein in plasma. F1 pups were assessed for transgene DNA in the liver. hFVIII human Factor VIII.

Male B6.129S6-*Rag2*^*tm1Fwa*^ N12 mice, which lack mature B and T lymphocytes, were administered a single intravenous injection of vehicle or 6 × 10^13^ vg/kg AAV5-hFVIII-SQ into the tail vein (Fig. 1). Eight mice who received vehicle and 16 mice who received AAV5-hFVIII-SQ were mated with naïve B6.129S6-*Rag2*^*tm1Fwa*^ N12 females 4 days after dosing, when the concentration of vector genomes was estimated to be at its peak in semen, based on the shedding profile of 7 participants who received AAV5-hFVIII-SQ (median time-to-peak, 1.29 weeks) [6]. Six mice who received vehicle and 34 mice who received AAV5-hFVIII-SQ were mated 37 days after dosing, when a full spermatogenesis cycle was expected to be complete [19–21]. Mice were randomly assigned to treatment groups. Animals were cohabitated for up to 9 days until mating occurred. F0 male mice mated early were euthanized 14 days post-dose, and F0 mice mated later were euthanized 50 days post-dose. Liver, testes, and blood samples were taken for molecular analyses. F1 pups had liver tissue harvested on postpartum day 21; pups found dead between days 1 and 20 were also collected for analysis.

Analysis of livers for transgene DNA was considered sufficient to assess the risk of germline transmission, as transgene DNA would be present in every cell of the F1 generation if a permanent modification had occurred in the F0 male germline. This is because transgene DNA integration into the host genome is required for stable germline transmission, given that nonintegrated transgene DNA (ie, episomal DNA) would be diluted and eliminated through the millions of cell divisions that occur during F1 embryogenesis and not be inherited through the germline. Therefore, if no transgene DNA was found in the livers of F1 mice, it can be assumed that transgene DNA did not cause inheritable F0 germline modifications that would lead to multigenerational germline transmission.

This study was performed in compliance with the principles of Good Laboratory Practice. All animal housing and study procedures complied with the *Guide of the Care and Use of Laboratory Animals* [22], and the protocol was approved by the Institutional Animal Care and Use Committee before initiation. Throughout the study period, viability checks and clinical observations, including body weight and general appearance, were made regularly. F0 and F1 mice were euthanized by carbon dioxide asphyxiation, after which tissue and blood samples were collected and preserved for molecular analyses.

### qPCR method

The tissue for qPCR analysis was flash frozen in liquid nitrogen and stored at −70 °C. DNA was extracted using the QIAsymphony DSP DNA Mini kit and Qiagen Reagent DX (Qiagen, Hilden, Germany). DNA samples were diluted to a uniform concentration of 0.2 µg/µL and stored at −20 °C. qPCR was performed to detect and measure transgene DNA present in tissue samples. A serial dilution of linearized FVIII-SQ plasmid was used as a positive control to generate a standard curve. A bulk master mix consisting of 1x TaqMan^®^ Environmental Master Mix (Sigma-Aldrich, St. Louis, MO), 900 nM FVIII-SQ forward and reverse primer, and 250 nM FVIII-SQ probe was used for qPCR (Supplementary Table 1). Primers and probe were validated to be specific for codon-optimized hFVIII-SQ and did not cross-react with genomic DNA murine FVIII sequences. Extracted study samples containing up to 1 µg DNA were run in triplicate along with negative and positive controls in a QuantStudio Flex Real-Time PCR instrument (Thermo Fisher Scientific, Waltham, MA) with standard cycling conditions. Fluorescence was read and DNA quantification was performed by the QuantStudio Flex Real-Time PCR system. Samples were considered negative only if no increase of fluorescence above the threshold was observed after 40 amplification cycles. The validated limit of detection with 95% detectability was 7.28 copies/reaction.

### Electrochemiluminesence assay

An antibody-based sandwich electrochemiluminescence (ECL) assay was performed to detect and quantitate hFVIII-SQ protein in plasma samples from F0 males. Whole blood samples were stored with a 3.2% sodium citrate anticoagulant solution and centrifuged within 30 min of collection to separate plasma, which was then stored at −70 °C. An affinity-purified mouse monoclonal antibody to the A2 domain of hFVIII conjugated to an extra-long chain, amine reactive N-hydrosulfosuccinimide biotin and an affinity-purified sheep polyclonal antibody conjugated to a ruthenium N-hydroxysulfosuccinimide tag were incubated together with plasma samples at room temperature with shaking to form complexes between hFVIII and the tagged antibodies. The mixture was then pipetted onto a plate blocked with 6% bovine serum albumin in Tris Buffered Saline with Tween^®^ 20 and incubated at room temperature with shaking to capture the biotin tags. Plates were triple washed and buffer-containing tripropylamine substrate was added to react electrochemically with the ruthenium. Antibody complexes containing hFVIII-SQ bound to both the biotinylated and ruthenylated antibodies generated ECL signals proportional to the amount of hFVIII-SQ present in the sample, which were detected and quantified by a Sector Imager 2400 plate reader (Meso Scale Discovery, Rockville, MA).

### Statistical methods

A novel 2-stage model was used to assess germline transmission from F0 males to F1 offspring, assuming two distinct events need to occur for a successful germline transmission: 1) F0 males that received AAV5-hFVIII-SQ acquire transgene DNA and transmit it to the F1 generation, and 2) F1 generation mice inherit and retain the transgene DNA. Under this statistical model, the probability of detecting germline transmission in at least 1 of the F1 offspring is given in the following equation.

1 $${{{{{{{\mathrm{Probability}}}}}}}}\;{{{{{{{\mathrm{of}}}}}}}}\;{{{{{{{\mathrm{detecting}}}}}}}}\;{{{{{{{\mathrm{transgene}}}}}}}}\;{{{{{{{\mathrm{DNA}}}}}}}}\;{{{{{{{\mathrm{in}}}}}}}} \ge 1\;{{{{{{{\mathrm{F1}}}}}}}}\;{{{{{{{\mathrm{offspring}}}}}}}} = 1 - \mathop {\prod}\nolimits_{i = 1}^N {[1 - P_1 + P_1 \ast (1 - P_2)^{m_i}]}$$

*P*_1_ is the probability that an F0 male acquires and transmits transgene DNA to its F1 offspring; *P*_2_ is the probability that the F1 offspring inherits and retains the transgene DNA; *P*_1_ * *P*_2_ is the total probability of germline transmission; *N* is the number of F0 males that had successful mating resulting in F1 offspring; and *m*_*i*_ is the number of F1 offspring from the *i*^*th*^ F0 male. Study power was calculated to estimate the number of F0 males and F1 pups necessary to assess the probability of germline transmission, assuming a certain level of risk. Based on the model, the number of mice required to be tested would increase as the study power increases or as the assumed probabilities of germline transmission decrease. After determining that no germline transmission had occurred experimentally, the formula used to calculate the study power was used to estimate the confidence level that the risk of germline transmission is lower than the assumptions made in the model, based on the actual number of F1 pups sired by each F0 male. Power and confidence calculations were performed in R v3.5.2 (R Foundation for Statistical Computing, Vienna, Austria).

## Results

### In vivo observations

Except for one male mouse who received AAV5-hFVIII-SQ, all F0 male mice dosed with vehicle or AAV5-hFVIII-SQ survived until scheduled euthanasia and appeared normal throughout the study. There were no clinical observations, changes in body weight, or necropsy observations that could be attributed to AAV5-hFVIII-SQ. The death of 1 mouse was considered unrelated to AAV5-hFVIII-SQ administration or protein expression, because it occurred 2 h after dosing, and there were no post-mortem indications. Although the study was designed to assess the risk of germline transmission and not the impact of AAV5-hFVIII-SQ on fertility or other reproductive parameters, there were no apparent changes in fertility parameters such as the number of pups born per mating in males that received AAV5-hFVIII-SQ relative to mice that received vehicle. In addition, there was no effect of AAV5-hFVIII-SQ on maternal behaviors or litter observations, as well as no clinical findings, body weight changes, or necropsy observations in F1 offspring.

### AAV5-hFVIII-SQ transduction in F0 males

**Fig. 2:**
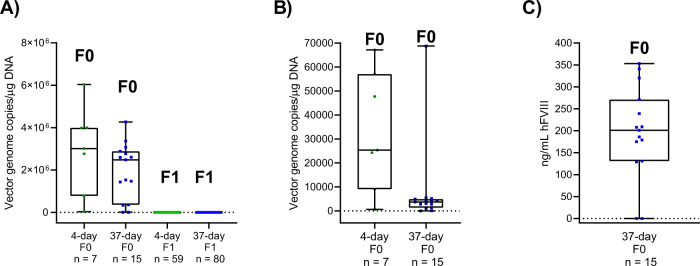
Levels of vector DNA and transgene protein. For the 22 F0 males who received AAV5-hFVIII-SQ and sired offspring after mating at 4 or 37 days post-dose, vector genome copy number in individual tissue samples from (**A**) liver or (**B**) testes, and (**C**) hFVIII-SQ protein plasma levels. Data are shown for the 22 F0 males who received AAV5-hFVIII-SQ and sired offspring and their 139 F1 male and female pups. No vector genome DNA was detected in any F0 male who received a vehicle injection. F0 males mated at 4 days post-dose were not evaluated for hFVIII protein presence. For the hFVIII protein assay, the LLOQ was 2.35 ng/mL; samples with protein levels below the LLOQ are shown as 0 ng/mL. hFVIII human Factor VIII, LLOQ lower limit of quantitation.

hFVIII-SQ DNA was detected in the livers of all F0 males who received AAV5-hFVIII-SQ mated at 4 days (*n* = 16) or 37 days post-dose (*n* = 34), consistent with successful gene transfer (Fig. 2A). In livers of these F0 males mated at 4 days, transgene DNA levels ranged from 14341 to 6036022 mean vector copies (vc)/μg DNA tested, and in F0 males mated at 37 days, liver transgene DNA levels ranged from 1725 to 4267407 mean vc/μg DNA tested. Transgene DNA was also detected at lower levels in the testes of all F0 males mated at 4 days (range, 138 to 84907 mean vc/μg DNA) and 37 days (33 to 68760 mean vc/μg DNA) (Fig. 2B). As expected, no F0 males dosed with vehicle control were positive for hFVIII-SQ DNA in their livers or testes. The plasma of 28 of 33 evaluated F0 males mated at 37 days had detectable hFVIII-SQ protein, ranging from 64 to 387 ng/mL (Fig. 2C). None of the six evaluated vehicle-dosed F0 mice mated at 37 days post-dose had hFVIII-SQ protein detected in plasma. The presence of hFVIII-SQ DNA in liver and testes and the corresponding detection of hFVIII-SQ protein in plasma further confirms that F0 males that received AAV5-hFVIII-SQ were successfully transduced and, therefore, had the theoretical potential for vertical transmission of AAV5-hFVIII-SQ to the next generation. By experimental design, hFVIII-SQ protein in plasma was not measured in F0 males mated at the earlier timepoint, because very low or no transgene expression was expected, given that euthanasia occurred soon after AAV5-hFVIII-SQ administration. However, F0 males mated at 4 days demonstrated high levels of hFVIII-SQ DNA in their livers and relatively lower levels of hFVIII-SQ DNA in their testes.

### Germline transmission in F1 mice

In total, 59 and 80 F1 male and female offspring from AAV5-hFVIII-SQ-dosed F0 males mated at 4 and 37 days, respectively, and 26 and 51 F1 males and females sired by vehicle-dosed F0 males mated at 4 and 37 days, respectively, were assessed for the presence of transgene DNA in the liver. No F1 mice sired by F0 males dosed with vehicle were positive for transgene DNA. More importantly, no transgene DNA was detected in the livers of F1 offspring of F0 males dosed with 6 × 10^13^ vg/kg AAV5-hFVIII-SQ (Fig. 2A).

**Fig. 3:**
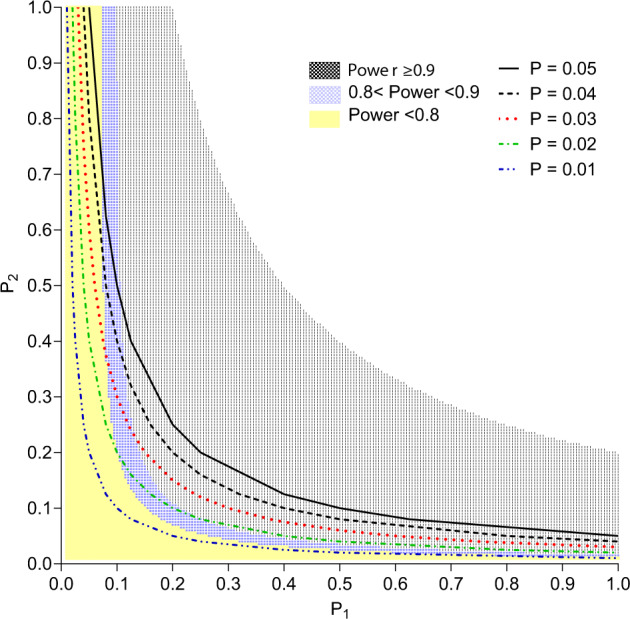
Contour plot showing the relationship between study confidence, *P*_1_, and *P*_2_ resulting from the 2-stage statistical model. Results were generated with Eq. 1. *P*_1_ is the probability an F0 male acquires and transmits transgene DNA to an F1 offspring. *P*_2_ is the probability an F1 offspring inherits and retains the transgene DNA. *P* = *P*_1_ * *P*_2_ is the overall probability of a germline transmission; lines represent combinations of *P*_1_ * *P*_2_ that would result in *P*, probability of germline transmission, between 0.01 and 0.05. The colored regions represent the combinations of *P*_1_ and *P*_2_, covering various ranges of study confidence, using the 22 F0 males who received AAV5-hFVIII-SQ and their combined 139 F1 offspring.

Based on the 2-stage statistical model that took into account 22 F0 males that received AAV5-hFVIII-SQ and the 139 F1 pups these males sired, there is 99.2% confidence that the risk of germline transmission is <5% (Table 1). A visual representation of possible confidence levels derived from the model, given various assumed values of *P*_1_ and *P*_2_, is presented in Fig. 3.

**Table 1:** Study confidence that ≥1 hFVIII-SQ germline transmission event would have been detected given assumed probabilities for the risk of germline transmission.

Overall probability of germline transmission (*P*)	*P*_*1*_	*P*_*2*_	Confidence/power
0.050	0.316	0.158	0.992
0.040	0.283	0.141	0.982
0.030	0.245	0.122	0.954
0.020	0.200	0.100	0.884
0.015	0.173	0.087	0.810

*P* = *P*_1_ * *P*_2_, where *P*_1_ is the assumed probability an F0 male acquires and transmits transgene DNA to an F1 offspring, and *P*_2_ is the assumed probability an F1 offspring inherits and retains the transgene DNA. Confidence values were calculated using Eq. 1 as stated in the Methods section and were based on the number of mice born to each of the 22 F0 males that received AAV5-hFVIII.

## Discussion

In this study, sexually mature male B6.129S6-*Rag2*^*tm1Fwa*^ N12 mice (F0) were dosed with 6 × 10^13^ vg/kg AAV5-hFVIII-SQ and subsequently bred with naïve females. Germline transmission potential was evaluated at two different mating time points following AAV5-hFVIII-SQ administration. Mating pairs were set up 4 days after dosing, when transgene DNA concentration was estimated to be highest in semen based on AAV5-hFVIII-SQ clinical data [6], and at 37 days after dosing, when the spermatogenesis cycle was expected to be complete in mice [19–21]. Delayed mating at 37 days after AAV5-hFVIII-SQ administration could potentially enable the transduction of early-stage spermatogonia that would then mature into sperm ready for fertilization at the time of mating. These two timepoints were chosen as they were estimated to have the highest potential for germline transmission. While transduction and genomic integration of transgene DNA could in theory occur at any stage of spermatogenesis, exhaustively testing every stage or through several spermatogenesis cycles is not feasible. While transgene DNA was detected in the testes of these F0 males, no instances of germline transmission were observed, as no transgene DNA was detected in any of the 139 pups (F1) they sired.

Our results are consistent with previous in vitro and in vivo studies that showed that germline transmission is unlikely following AAV-mediated gene transfer. For instance, exposing mouse sperm to high concentrations of AAV2 vectors did not result in germline transmission when followed by in vitro fertilization [23]. Previous in vivo approaches to assess the risk of germline transmission in rats, mice, or rabbits included the delivery of AAV vectors systemically or directly into the gonads, followed by an assessment of gonadal tissue or gametes for the presence of transgene DNA [8, 10, 12, 16, 23]. However, studies examining gametes are typically only feasible with a relatively small number of animals, and visualization of transgene DNA using DNA-hybridization techniques can only be performed in a fraction of the total gamete population, particularly when assessing the male reproductive tract. In other animal studies, AAV vectors were administered systemically to males, which were then mated with naïve females, and the resulting offspring of such pairings were then evaluated for the presence of transgene DNA [11, 24, 25]. Unlike the current study, most of these studies were performed without power calculations or other statistical considerations to provide a confidence level to determine the probability of germline transmission.

To incorporate statistical considerations into the experimental design and data interpretation, we developed a novel 2-stage statistical model that assumed two distinct events needed to occur for successful germline transmission: (1) F0 males that received AAV5-hFVIII-SQ acquired transgene DNA and transmitted it to the F1 generation, and (2) F1 generation mice inherited and retained the transgene DNA. This statistical model was initially used to perform a power analysis calculation to identify the minimal number of F0 and F1 mice necessary to detect at least one successful germline transmission event, based on hypothetical probabilities assigned to the two transmission steps defined above. Importantly, the minimal number of F0 progenitors utilized, as determined by the model, ensured that the contribution of each F0 male to the F1 generation was appropriately calculated and avoided underestimating germline transmission risk by using too few males. Once the experiments were completed, the data were analyzed by the model to establish a confidence level supported by the actual results. Given that no AAV5-hFVIII-SQ DNA was detected in F1 offspring, the model supports a 99.2% confidence that the risk of germline transmission of AAV5-hFVIII-SQ is below 5%. This novel method provides a level of statistical confidence in our results that has previously been lacking from studies of germline transmission, as an infinite number of mice would be required to prove that germline transmission does not occur following AAV5-hFVIII-SQ therapy, or any other gene therapy.

The low potential for germline transmission observed in this study was expected, given the engineered design of AAV vectors, including their inability to replicate [4]. Genome integration is likely to be a required step for successful AAV-mediated germline transmission to occur. Relatively low levels of genome integration cannot be ruled out, as shown in a number of published reports; [26–31] however, given the largely episomal and nonreplicating nature of transduced AAV vector DNA [32–41], even a rare event of episomal germline transmission would be highly unlikely to result in multigenerational, stable transgene inheritance. Overall, our results show that germline transmission of AAV5-hFVIII-SQ is highly unlikely.

## Supplementary information

Supplemental material

## Data Availability

Materials and protocols will be distributed to qualified scientific researchers for non-commercial, academic purposes. The AAV5-hFVIII-SQ vector and the AAV5-hFVIII-SQ vector sequence are part of an ongoing development program, and they will not be shared.
