# Supplementary material for: Lack of germline transmission in male mice following a single intravenous administration of AAV5-hFVIII-SQ gene therapy
Source: Gene Ther. 2022 Feb 7;30(7-8):581–6. doi: 10.1038/s41434-022-00318-5 (PMC10457182; doi:10.1038/s41434-022-00318-5)
Supplement: Supplementary file 1 — Supplemental material [file 41434_2022_318_MOESM1_ESM.docx]

**SUPPLEMENTAL MATERIAL**

**Supplemental Table 1.** Primers and probes for qPCR

| **Primer/Probe** | **Sequence** |
| --- | --- |
| hFVIII-SQ forward | ATG CAC AGC ATC AAT GGC TA |
| hFVIII-SQ reverse | CCA TCT TGT GCT TGA AGG TG |
| hFVIII-SQ probe | FAM-CCT GAG CAT TGG GGC CCA GA-BHQ1 |

Primers were sourced from Sigma-Aldrich (St. Louis, MO). FVIII, Factor VIII.
